# Supplementary material for: Novel Driver Strength Index highlights important cancer genes in TCGA PanCanAtlas patients
Source: PeerJ. 2022 Aug 11;10:e13860. doi: 10.7717/peerj.13860 (PMC9375969; doi:10.7717/peerj.13860)
Supplement: Supplemental Information 2 [file peerj-10-13860-s002.zip › COHORTS/genes plots/cohorts/2021_8_16_14_9_distribution_cohorts_UVM.pdf]

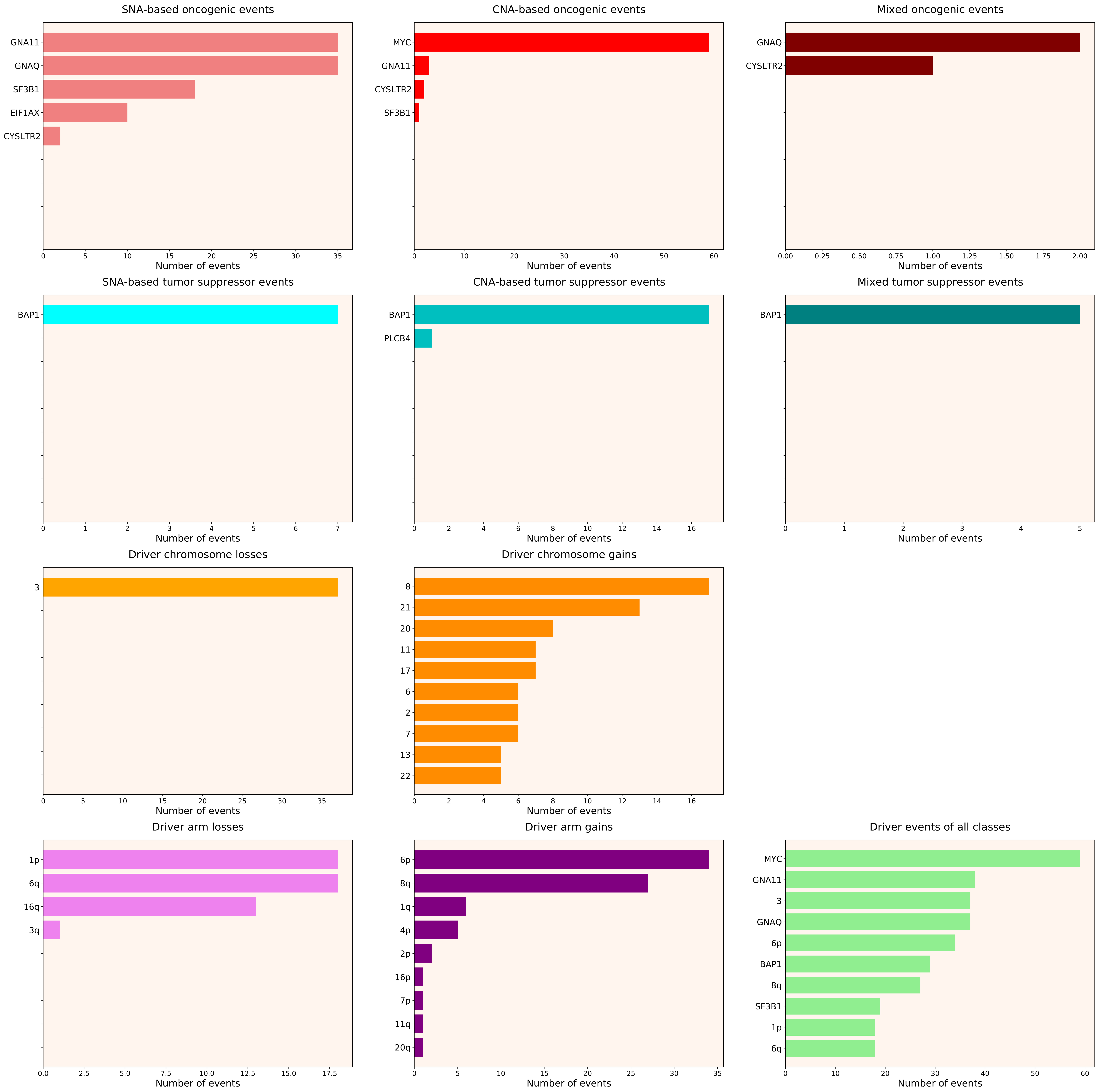
SNA-based oncogenic events
GNA11
GNAQ
SF3B1
EIF1AX
CYSLTR2
0 5 10 15 20 25 30 35
Number of events
CNA-based oncogenic events
MYC
GNA11
CYSLTR2
SF3B1
0 10 20 30 40 50 60
Number of events
Mixed oncogenic events
GNAQ
CYSLTR2
0.00 0.25 0.50 0.75 1.00 1.25 1.50 1.75 2.00
Number of events
SNA-based tumor suppressor events
BAP1
0 1 2 3 4 5 6 7
Number of events
CNA-based tumor suppressor events
BAP1
PLCB4
0 2 4 6 8 10 12 14 16
Number of events
Mixed tumor suppressor events
BAP1
0 1 2 3 4 5
Number of events
Driver chromosome losses
3
0 5 10 15 20 25 30 35
Number of events
Driver chromosome gains
8
21
20
11
17
6
2
7
13
22
0 2 4 6 8 10 12 14 16
Number of events
Driver arm losses
1p
6q
16q
3q
0.0 2.5 5.0 7.5 10.0 12.5 15.0 17.5
Number of events
Driver arm gains
6p
8q
1q
4p
2p
16p
7p
11q
20q
0 5 10 15 20 25 30 35
Number of events
Driver events of all classes
MYC
GNA11
3
GNAQ
6p
BAP1
8q
SF3B1
1p
6q
0 10 20 30 40 50 60
Number of events
